# Patterns and Perceptions of Smartphone Use Among Academic Neurologists in the United States: Questionnaire Survey

**DOI:** 10.2196/22792

**Published:** 2020-12-24

**Authors:** William Zeiger, Scott DeBoer, John Probasco

**Affiliations:** 1 Department of Neurology University of California, Los Angeles School of Medicine Los Angeles, CA United States; 2 Medstar Franklin Square Medical Center Baltimore, MD United States; 3 Department of Neurology Georgetown University Washington, DC United States; 4 Department of Neurology Johns Hopkins University School of Medicine Baltimore, MD United States

**Keywords:** smartphone, mobile phone, mobile apps, mobile health, neurology, neurologic exam, physical exam

## Abstract

**Background:**

Smartphone technology is ubiquitous throughout neurologic practices, and numerous apps relevant to a neurologist’s clinical practice are now available. Data from other medical specialties suggest high utilization of smartphones in routine clinical care. However, the ways in which these devices are used by neurologists for patient care–related activities are not well defined.

**Objective:**

This paper aims to characterize current patterns of smartphone use and perceptions of the utility of smartphones for patient care–related activities among academic neurology trainees and attending physicians. We also seek to characterize areas of need for future app development.

**Methods:**

We developed a 31-item electronic questionnaire to address these questions and invited neurology trainees and attendings of all residency programs based in the United States to participate. We summarized descriptive statistics for respondents and specifically compared responses between trainees and attending physicians.

**Results:**

We received 213 responses, including 112 trainee and 87 attending neurologist responses. Neurology trainees reported more frequent use of their smartphone for patient care–related activities than attending neurologists (several times per day: 84/112, 75.0% of trainees; 52/87, 59.8% of attendings; *P*=.03). The most frequently reported activities were internet use, calendar use, communication with other physicians, personal education, and health care–specific app use. Both groups also reported regular smartphone use for the physical examination, with trainees again reporting more frequent usage compared with attendings (more than once per week: 35/96, 36.5% of trainees; 8/58, 13.8% of attendings; *P*=.03). Respondents used their devices most commonly for the vision, cranial nerve, and language portions of the neurologic examination. The majority of respondents in both groups reported their smartphones as “very useful” or “essential” for the completion of patient care–related activities (81/108, 75.0% of trainees; 50/83, 60.2% of attendings; *P=*.12). Neurology trainees reported a greater likelihood of using their smartphones in the future than attending neurologists (“very likely”: 73/102, 71.6% of trainees; 40/82, 48.8% of attendings; *P*=.005). The groups differed in their frequencies of device usage for specific patient care–related activities, with trainees reporting higher usage for most activities. Despite high levels of use, only 12 of 184 (6.5%) respondents reported ever having had any training on how to use their device for clinical care. Regarding future app development, respondents rated vision, language, mental status, and cranial nerve testing as potentially being the most useful to aid in the performance of the neurologic examination.

**Conclusions:**

Smartphones are used frequently and are subjectively perceived to be highly useful by academic neurologists. Trainees tended to use their devices more frequently than attendings. Our results suggest specific avenues for future technological development to improve smartphone use for patient care–related activities. They also suggest an unmet need for education on effectively using smartphone technology for clinical care.

## Introduction

Smartphones are a ubiquitous presence on hospital wards. Ownership among physicians is nearly universal [1-8], with most regularly using their devices for phone calls, texting, email, and internet [4,6]. Rising rates of usage among physicians have been paralleled by a proliferation of health care–specific smartphone apps. These are generally rated as useful for clinical practice, especially for providing quick access to references and clinical score calculators [1,3-6]. As smartphone technology improves, clinical uses for these devices continue to expand. An increasing number of these devices are being used in creative ways to directly augment and improve bedside patient diagnosis and care. Smartphones can be used for vital signs [9], telemetry monitoring [10], ambulatory electroencephalography (EEG) [11], and even as portable ultrasounds [12], otoscopes [13], and ophthalmoscopes [14].

There are now many neurology-specific smartphone apps, with an emphasis on everything from anatomy to localization, reference materials, education, and documentation [15]. Mobile photo and video capture capabilities can help characterize intermittent symptoms such as seizures [16,17] or allow for remote telemedicine evaluation of acute stroke [18]. For patient monitoring in the home environment, there are symptom trackers (eg, headache diaries [19]) and apps to track functional impairments related to Parkinson disease [20], multiple sclerosis [21], and dementia [22,23]. To assist at the bedside, smartphone apps can now be found to evaluate everything from visual function [24,25] to tremors [26], gait speed [27], joint range of motion [28-30], and spinal deformities [31]. Despite the promise of smartphone technology, little is known about the current use of smartphones by neurologists in patient care or about areas of need to guide future app development. Therefore, we designed a survey to characterize current practice patterns of smartphone use among attending academic neurologists and neurology trainees. We also sought to identify parts of the neurologic examination that neurologists find to be most in need of adjunctive technological innovations.

## Methods

The study was approved by the Johns Hopkins University School of Medicine Institutional Review Board. An initial draft questionnaire was developed by the authors and was subsequently refined and validated through 2 focus groups consisting of a total of 4 residents and 3 attendings from the Department of Neurology at the Johns Hopkins University School of Medicine. Focus group participants provided direct oral and written feedback regarding the questionnaire length and subject areas, as well as the clarity, response options, and relevance of items in the questionnaire about their experience using smartphones. Patient care–related activities were clarified to include “communication with or about patients, clinical documentation, physical examination, accessing clinical or reference information, and healthcare specific mobile applications.” The final questionnaire was distributed electronically using Qualtrics software.

A letter with an anonymous link to the final 31-item questionnaire was emailed to all program directors and coordinators of academic neurology residency training programs (154 programs in total) in the United States in the spring of 2018. Follow-up reminder emails were sent 1 and 2 months later, and data collection was closed 3 months after the initial invitation. We did not solicit or receive feedback from programs about whether they had distributed the survey to physicians at their program, and as a result, we were unable to calculate a complete response rate for the questionnaire. On the first page of the questionnaire, participants were told the purpose of the survey and the estimated length of time to complete the survey (10 minutes) and were informed that participation was completely voluntary and that participation in the survey would serve as consent to have responses included in the study. Respondents could leave questionnaire items incomplete. No personal or identifying data were collected or stored about respondents. We did not collect information about the institutions to which respondents belonged nor did we attempt to validate self-reported usage data with data logs from respondents’ smartphones.

Data were analyzed using MATLAB (MathWorks) and R (R Foundation for Statistical Computing). Questionnaires with incomplete data were included in the analysis. Results are presented with the total number of respondents for each questionnaire item. Primary analysis was done with chi-square tests. When expected counts were low (<5), response categories were binned. When response categories could not be logically binned, a Fisher exact test was used. A threshold for statistical significance of 0.05 was used. Follow-up 2 × 2 contingency tables were created for post hoc testing of individual response categories with Bonferroni correction. For matrix table items with Likert-type scales, data were compared using the Wilcoxon rank sum test with Bonferroni correction.

## Results

**Figure 1:**
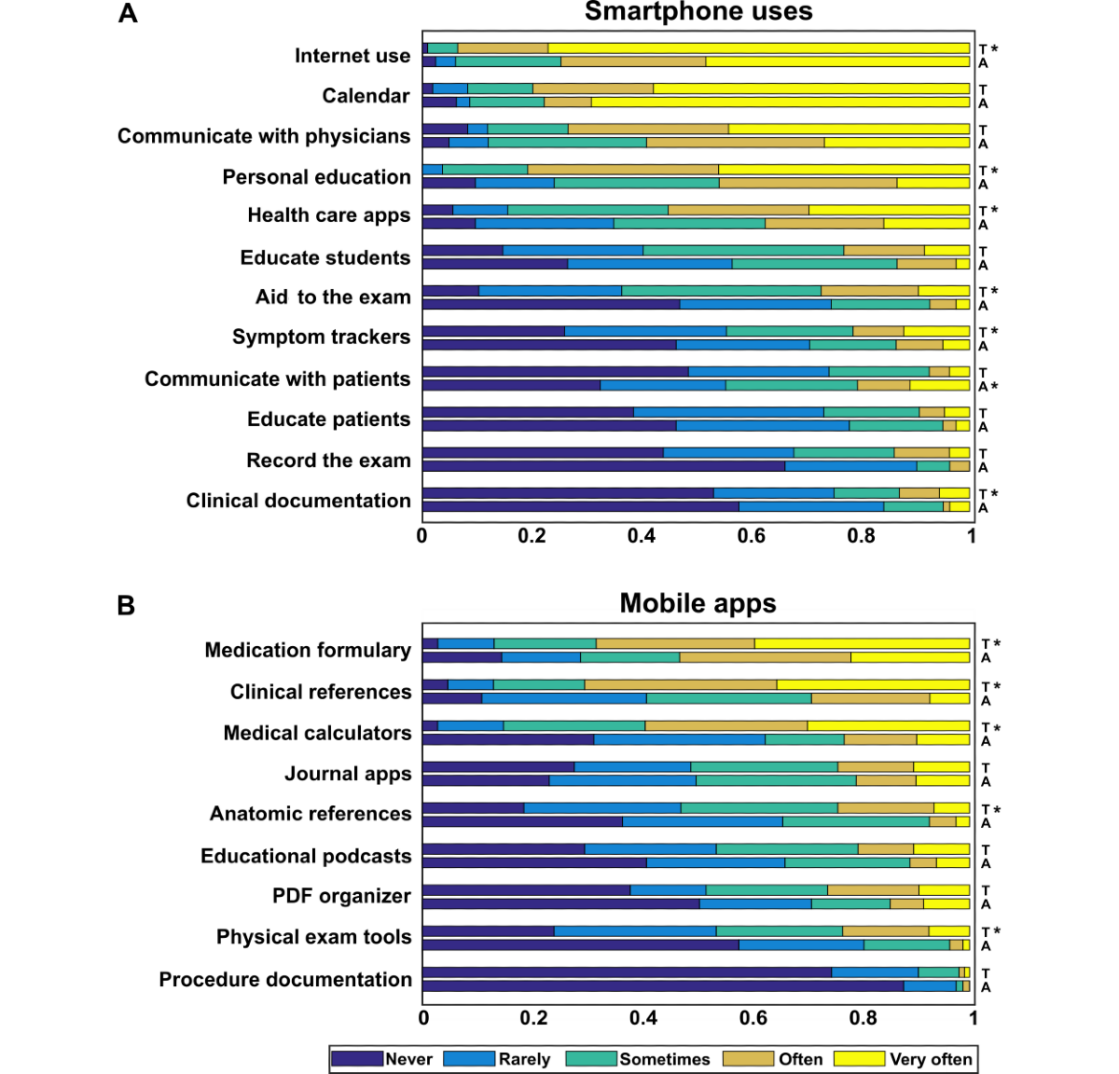
Frequency of smartphone and mobile app use. (A) Respondents were asked, “How frequently do you use your smartphone and/or tablet for the following patient care related activities?” (B) Respondents were asked, “How frequently do you use the following types of mobile applications?” A: attending physicians; T: trainees. *Significantly greater usage for the indicated group compared with the other, with Bonferroni adjusted *P*<.05.

A total of 213 neurologists responded to the questionnaire, all of whom owned smartphones. We estimate our response rate was about 4% for trainees, based on 112 trainee responses and a total of 2797 neurology residents and fellows in 2018 [32]. Demographics are presented in Table 1. Overall, smartphone use for patient care–related activities was high. The majority of respondents reported using their smartphone several times per day, with trainees reporting more frequent usage (84/112, 75.0% of trainees and 52/87, 59.8% of attending physicians; *P*=.03) and longer duration of use per day (median of 31-50 minutes for trainees and 11-30 minutes for attending physicians; *P*=.02) (Table 2). A variety of specific patient care–related activities for which respondents used their devices were surveyed, with the most frequently reported activities being internet use, calendar use, communication with other physicians, personal education, and health care–specific app use (Figure 1). Trainees reported greater smartphone usage patterns for most activities. The specific mobile apps used by each group are summarized in Figure 1. The majority of respondents in both groups reported that their smartphones were “very useful” or “essential” for the completion of patient care activities (Table 2).

**Table 1:** Demographics of survey respondents.

Characteristics		All	Attending	Trainee	*P* value
Sex (female), n/N (%)		93/199 (46.7)	37/87 (42.5)	56/112 (50.0)	.36
**Age (years), median**		30-34	40-49	30-34	<.001
	<30, n/N (%)^a^	36/198 (18.2)	1/87 (1.1)	35/111 (31.5)	
	30-34, n/N (%)^a^	66/198 (33.3)	6/87 (6.9)	60/111 (54.1)	
	35-39, n/N (%)	32/198 (16.2)	17/87 (19.5)	15/111 (13.5)	
	40-49, n/N (%)^a^	29/198 (14.6)	29/87 (33.3)	0/111 (0.0)	
	50-59, n/N (%)^a^	15/198 (7.6)	14/87 (16.1)	1/111 (0.9)	
	>60, n/N (%)^a^	20/198 (10.1)	20/87 (23.0)	0/111 (0.0)	
PGY^b^, median		N/A^c^	N/A	3	N/A
Years in practice, median		N/A	14	N/A	N/A

^a^Individual response category was found to be significant upon post hoc testing with Bonferroni correction.

^b^PGY: postgraduate year.

^c^N/A: not applicable.

**Figure 2:**
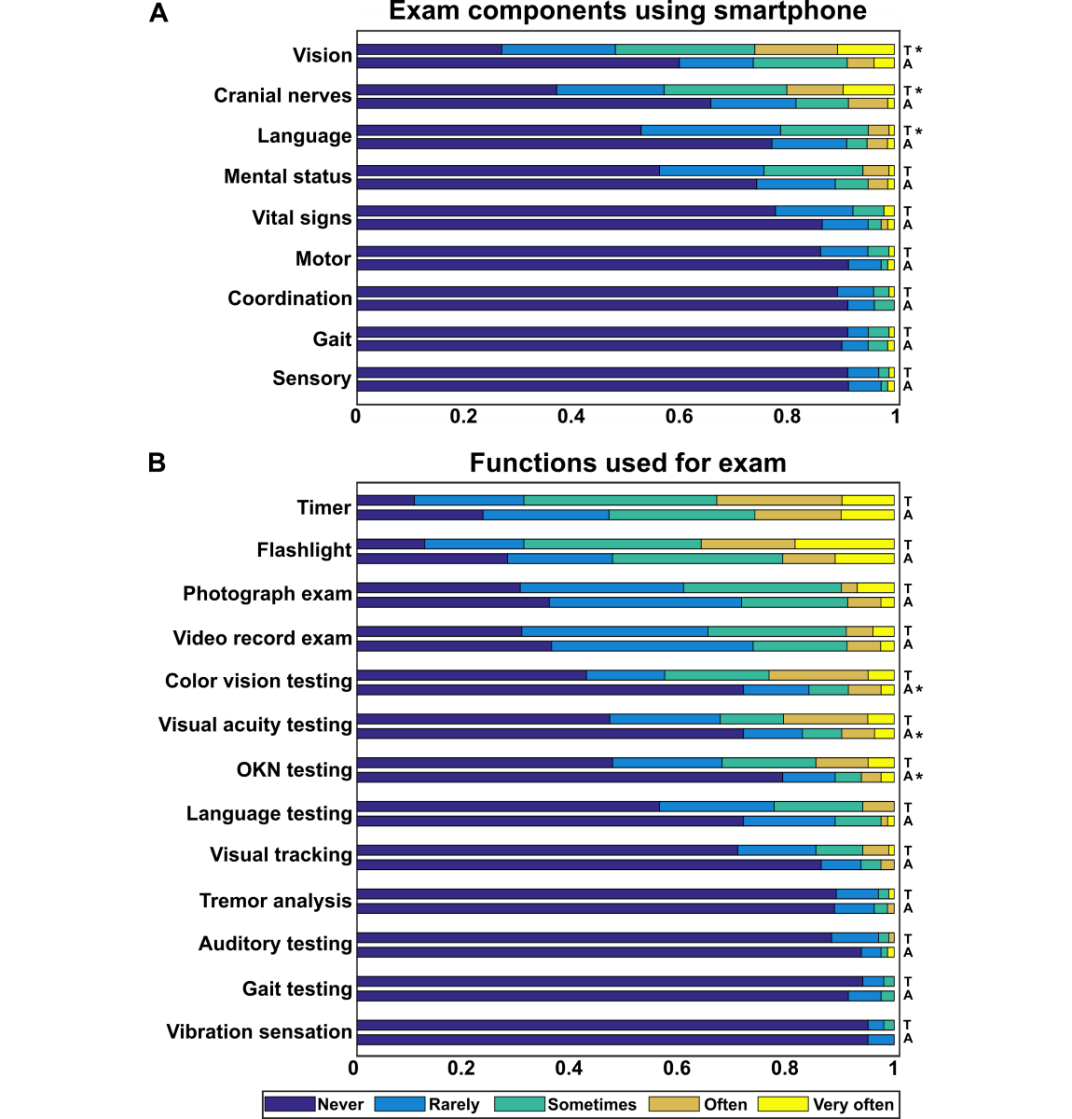
Frequency of smartphone use for the examination. (A) Respondents were asked, “How frequently do you utilize your smartphone and/or tablet for the following parts of the neurologic examination?” (B) Respondents were asked, “How frequently do you use your smartphone and/or tablet for each of the following functions when performing the physical examination?” A: attending physicians; OKN: optokinetic nystagmus; T: trainees. *Significantly greater usage for the indicated group compared with the other, with Bonferroni adjusted *P*<.05.

Respondents were also surveyed regarding current usage of their devices as an aid to the performance of the neurologic examination. Most respondents said they had used their smartphone as an aid to the examination, with more trainees having done so compared to attending physicians (97/108, 89.8% trainees vs 58/83, 69.9% attending physicians; *P*<.001) (Table 2). Frequency of smartphone usage as an aid to the neurologic examination was lower than for overall use, with a median response in both groups of “2-3 times a month.” Respondents used their devices most commonly for the vision, cranial nerve, and language portions of the neurologic examination, with trainees reporting more frequent usage compared with attending physicians (Figure 2). The specific smartphone functions most frequently used are summarized in Figure 2. Very few respondents reported having ever received any instruction in the use of a smartphone as an aid to the neurologic examination (Table 2).

**Figure 3:**
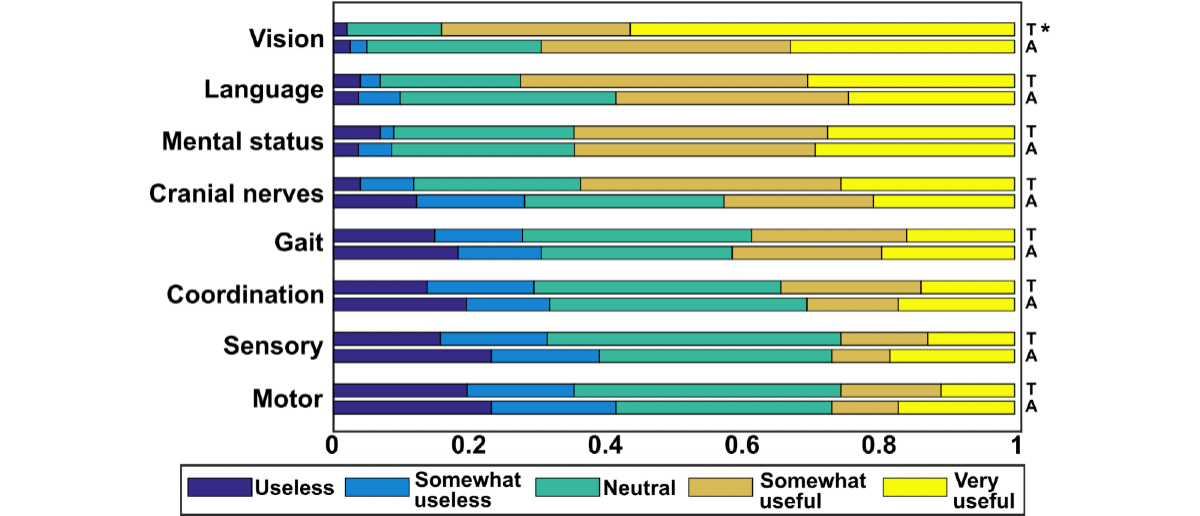
Perceived utility of potential new smartphone apps. Respondents were asked, “Imagine that a new mobile application was developed to aid in the performance of the neurologic examination. Please rank how useful it would be to have an application that could enhance the performance of each area of testing listed below.” A: attending physicians; T: trainees. *Significantly greater usage for the indicated group compared with the other, with Bonferroni adjusted *P*<.05.

Finally, respondents were asked about their expectations regarding future smartphone use. The majority of respondents reported a high likelihood (“likely” or “very likely”) of using their devices for patient care–related activities in the future, with trainees reporting higher likelihood (*P*=.005) (Table 2). Subjective likelihood of future device use as an aid to the neurologic examination was also high, with trainees reporting greater likelihoods (median response for trainees was “likely” vs “somewhat likely” for attending physicians; *P*=.05) (Table 2). When asked to imagine that a new mobile app was developed to aid in the performance of the neurologic examination, respondents reported the greatest potential utility for apps enhancing vision, language, and mental status testing (Figure 3). Respondents almost universally expected future use of their devices to be at similar or greater levels than current usage (Table 2).

Given that we found several differences between attending physicians and trainees, we wondered how much of this effect could have been driven by age rather than training status. Therefore, we conducted a subgroup analysis for respondents in the age range with the greatest overlap between attending physicians and trainees (35-39 years). In this age range, we did not find any significant differences between groups for any of the items reported in Table 2. While our study is not sufficiently powered for this type of subgroup analysis, these results do suggest that differences between trainees and attending physicians may be attributable to age rather than training status per se.

**Table 2:** Patterns of current smartphone usage and predicted future usage.

Usage		All	Attending	Trainee	*P* value
**Frequency of use, median**		Several times a day	Several times a day	Several times a day	.03
	Once a week or less, n/N (%)	18/199 (9.0)	13/87 (14.9)	5/112 (4.5)	
	2-3 times a week, n/N (%)	19/199 (9.5)	11/87 (12.6)	8/112 (7.1)	
	Once or twice a day, n/N (%)	26/199 (13.1)	11/87 (12.6)	15/112 (13.4)	
	Several times a day, n/N (%)	136/199 (68.3)	52/87 (59.8)	84/112 (75.0)	
**Duration of use (min), median**		11-30 min	11-30 min	31-50 min	.02
	<10, n/N (%)^a^	34/199 (17.1)	22/87 (25.3)	12/112 (10.7)	
	11-30, n/N (%)	69/199 (34.7)	32/87 (36.8)	37/112 (33.0)	
	31-50, n/N (%)	29/199 (14.6)	11/87 (12.6)	18/112 (16.1)	
	>50, n/N (%)	67/199 (33.7)	22/87 (25.3)	45/112 (40.2)	
**Number of apps, median**		<5	<5	<5	.03
	<5, n/N (%)^a^	121/198 (61.1)	62/86 (72.1)	59/112 (52.7)	
	6-10, n/N (%)^a^	50/198 (25.3)	13/86 (15.1)	37/112 (33.0)	
	11-15, n/N (%)	15/198 (7.6)	6/86 (7.0)	9/112 (8.0)	
	>15, n/N (%)	12/198 (6.1)	5/86 (5.8)	7/112 (6.3)	
Ever used for examination (yes), n/N (%)		155/191 (81.2)	58/83 (69.9)	97/108 (89.8)	<.001
**Frequency of use for examination, median**		2-3 times a month	2-3 times a month	2-3 times a month	.03
	Less than once a month, n/N (%)	40/154 (26.0)	18/58 (31.0)	22/96 (22.9)	
	Once a month, n/N (%)	19/154 (12.3)	8/58 (13.8)	11/96 (11.5)	
	2-3 times a month, n/N (%)	32/154 (20.8)	13/58 (22.4)	19/96 (19.8)	
	Once a week, n/N (%)	20/154 (13.0)	11/58 (19.0)	9/96 (9.4)	
	2-3 times a week, n/N (%)	27/154 (17.5)	7/58 (12.1)	20/96 (20.8)	
	Daily, n/N (%)	16/154 (10.4)	1/58 (1.7)	15/96 (15.6)	
**Usefulness, median**		Very useful	Very useful	Very useful	.12
	Not useful at all, n/N (%)	12/191 (6.3)	8/83 (9.6)	4/108 (3.7)	
	Minimally useful, n/N (%)	48/191 (25.1)	25/83 (30.1)	23/108 (21.3)	
	Very useful, n/N (%)	85/191 (44.5)	34/83 (41.0)	51/108 (47.2)	
	Essential, n/N (%)	46/191 (24.1)	16/83 (19.3)	30/108 (27.8)	
Ever received instruction (yes), n/N (%)		12/184 (6.5)	2/82 (2.4)	10/102 (9.8)	.09
**Likelihood of future use, median**		Very likely	Likely	Very likely	.005
	Very unlikely, n/N (%)	2/184 (1.1)	2/82 (2.4)	0/102 (0.0)	
	Unlikely, n/N (%)	1/184 (0.5)	1/82 (1.2)	0/102 (0.0)	
	Somewhat unlikely, n/N (%)	3/184 (1.6)	3/82 (3.7)	0/102 (0.0)	
	Undecided, n/N (%)	9/184 (4.9)	7/82 (8.5)	2/102 (2.0)	
	Somewhat likely, n/N (%)	14/184 (7.6)	8/82 (9.8)	6/102 (5.9)	
	Likely, n/N (%)	42/184 (22.8)	21/82 (25.6)	21/102 (20.6)	
	Very likely, n/N (%)	113/184 (61.4)	40/82 (48.8)	73/102 (71.6)	
**Likelihood of future use for examination, median**		Likely	Somewhat likely	Likely	.05
	Very unlikely, n/N (%)	8/184 (4.3)	6/82 (7.3)	2/102 (2.0)	
	Unlikely, n/N (%)	6/184 (3.3)	4/82 (4.9)	2/102 (2.0)	
	Somewhat unlikely, n/N (%)	9/184 (4.9)	7/82 (8.5)	2/102 (2.0)	
	Undecided, n/N (%)	25/184 (13.6)	14/82 (17.1)	11/102 (10.8)	
	Somewhat likely, n/N (%)	27/184 (14.7)	11/82 (13.4)	16/102 (15.7)	
	Likely, n/N (%)	44/184 (23.9)	17/82 (20.7)	27/102 (26.5)	
	Very likely, n/N (%)	65/184 (35.3)	23/82 (28.0)	42/102 (41.2)	
**Frequency of future use, median**		More	More	Same or more	.37
	Never, n/N (%)	2/184 (1.1)	1/82 (1.2)	1/102 (1.0)	
	Less than current usage, n/N (%)	1/184 (0.5)	0/82 (0.0)	1/102 (1.0)	
	The same as current usage, n/N (%)	80/184 (43.5)	31/82 (37.8)	49/102 (48.0)	
	More than current usage, n/N (%)	101/184 (54.9)	50/82 (61.0)	51/102 (50.0)	

^a^Individual response category was found to be significant upon post hoc testing with Bonferroni correction.

## Discussion

As smartphone technologies improve, neurologists frequently use their mobile devices for patient care–related activities. Standard items from the neurologist’s tool kit, such as a wristwatch and penlight, can easily be replaced with basic smartphone functionalities. Apps supplanting more advanced testing are rapidly being incorporated as well, including apps for visual acuity and color vision testing [24,25], tremor analysis [26], ophthalmoscopy [14], cognitive testing [23], and EEG [11]. Our results, which demonstrate ubiquitous and frequent usage of smartphones by neurologists, are in broad agreement with data across other specialties. For example, a 2011 survey distributed to all residents, fellows, and attending physicians participating in Accreditation Council for Graduate Medical Education training programs found that 85% of respondents owned smartphones and 56% used apps in their clinical practice [1]. Similar or greater usage has been found among residents in internal medicine [4,6], pediatrics [2], neurosurgery [7], obstetrics [8], urology [5], and radiation oncology [3] training programs, both in the United States and abroad. We found that the majority of neurologists use their devices for patient care–related activities several times per day, including for communication, educational activities, and health care–specific apps, as well as at the bedside as an aid in the performance of the physical examination. Smartphones were used for multiple portions of the neurologic examination, most commonly for vision, cranial nerve, language, and mental status testing.

These practice patterns are unlikely to be transient, as most respondents in this study predicted high likelihood of future smartphone use, including as an aid to the neurologic examination. We anticipate that the use of smartphone apps in neurologic practice will continue to grow, as trainees use their devices more frequently than attending physicians across a range of smartphone apps and functions. Indeed, neurology trainees tended to use their devices more frequently both for general patient care–related activities and as an aid to the performance of the physical examination. These trends held true when examining specific smartphone apps and functions, with trainees tending to report higher usage for most categories, with the exception of communication with patients. Trainees also reported higher likelihood of future use, though subjective usefulness was similar between trainees and attending physicians. Although not powered for a subgroup analysis, responses were similar between trainees and attending physicians aged 35 to 39 years. This suggests that age may be a significant factor in the overall differences between these groups, with younger neurologists using their devices more, which further emphasizes the likelihood that smartphone use in neurologic practice will continue to grow.

In addition to changes driven by the demographics of neurologists entering the workforce, we expect other factors may increase reliance on smartphone technologies for patient care. The SARS-CoV-2 pandemic has led to a dramatically increased reliance by neurologists on telehealth technologies for remote care delivery [33]. As telehealth grows as a method of care delivery, there will be an increasing need for apps that neurologists and their patients can use to augment their telehealth encounter. Although conducted prior to the pandemic, this survey provides end user insight into areas of need that may guide smartphone app development for neurologic telehealth care. Smartphone apps that augment in-person vision, language, and mental status testing could also be designed for patients to use on their own devices during a telehealth visit, bringing the examination tool kit from the clinic to the patient.

Although a large majority of neurologists use their devices, almost none have had any education on how to do so effectively for clinical practice. The development of such a curriculum could have several benefits, including greater use, increased efficiency, expanded access, improved subjective utility, and potentially, encouragement to spur the next generation of app development. On the other hand, such a curriculum could address mitigation of the negative effects of smartphones, such as impaired sleep [34], distractibility [35,36], burnout [37], and confidentiality issues [38]. Education should also help physicians vet mobile technologies for incorporation into their practice, as the validity of smartphone apps for patient care is not always well established. Highlighting this fact, a recent review of apps related to emergency medicine found only a small percentage of apps to be clinically relevant [39]. Indeed, most apps and functionalities available today have not yet been well studied. The US Food and Drug Administration has recently recognized the unique challenges posed by mobile medical apps and has begun issuing policy guidance for manufacturers and distributors of these apps [40,41]. However, a comprehensive framework allowing patients and providers to easily evaluate mobile medical apps remains lacking [42].

This study was limited in several ways. The questionnaire was distributed to academic neurology training programs, so these findings may not be generalizable to private practice or nonacademic hospital settings. Participation was voluntary and our sample may have been biased toward neurologists with an interest in technology, who might have been more likely to respond to the questionnaire. All respondents were active smartphone users, and this might have resulted in an overestimation of the frequency of use or subjective usefulness, though based on our own experience, this seems unlikely. In addition, all smartphone use data were self-reported, and we did not validate these with objective data use logs. Finally, although our total number of respondents was large, our overall response rate was likely low. Given a total of 2797 neurology residents and fellows in 2018 [32] and 112 trainee respondents, we estimate that our response rate was approximately 4% for trainees.

In summary, smartphones are a valuable tool in academic neurology not only for communication but also for education and practice. These devices now feature in the neurologist’s equipment bag alongside the reflex hammer and tuning fork. Smartphone-owning neurologists expect to continue using their devices in the future. There is opportunity for further refinement of these devices for neurologic practice, limited only by our creativity in the use of features and the development of associated tools, scales, and apps. We anticipate that these ubiquitous handheld devices will in time prove invaluable to the diagnosis and treatment of patients with neurologic disease.

## Abbreviations

EEG: electroencephalography
